# Persistent SARS-CoV-2 RNA Positive in Feces but Negative in Breastmilk: A Case Report of COVID-19 in a Breastfeeding Patient

**DOI:** 10.3389/fmed.2020.562700

**Published:** 2020-12-02

**Authors:** Huikuan Chu, Jing Li, Jingjing Yan, Tai Bai, Bernd Schnabl, Li Zou, Ling Yang, Xiaohua Hou

**Affiliations:** ^1^Division of Gastroenterology, Tongji Medical College, Union Hospital, Huazhong University of Science and Technology, Wuhan, China; ^2^Department of Medicine, University of California, San Diego, La Jolla, CA, United States; ^3^Department of Obstetrics & Gynecology, Tongji Medical College, Union Hospital, Huazhong University of Science and Technology, Wuhan, China

**Keywords:** breastfeeding transmission, breastfeeding, fecal-oral transmission, SARS-CoV-2 RNA, COVID-19

## Abstract

COVID-19 is a pandemic infectious disease. Whether SARS-CoV-2 was transmitted through breast milk is unknown. Here, we report a breastfeeding woman with COVID-19 presenting with gastrointestinal symptoms and persistent SARS-CoV-2 RNA positivity in both her oropharyngeal swabs and feces, but negativity in her breastmilk. After appearance of serum SARS-CoV-2-IgG, she began to bottle feed her baby with breastmilk without transmission. This report facilitates the understanding of breastfeeding-related risks in COVID-19.

## Introduction

In December 2019, there was an outbreak of severe acute respiratory syndrome coronavirus 2 (SARS-CoV-2), which is the causative pathogen of Corona Virus Disease 2019 (COVID-19), in Wuhan, Hubei Province, China. COVID-19 spread rapidly from Wuhan to other areas in the world and there were 85,320 confirmed cases in China and 11,327,790 confirmed cases worldwide until July 6 (1, 2). COVID-19 has been considered as a public health emergency of international concern by the World Health Organization (WHO) as it seriously threatens human health and quality of life.

COVID-19 mainly affects the lower respiratory tract and manifests as pneumonia in humans, and is mainly transmitted among subjects by respiratory droplets and contact route (3). Besides, a small cluster of patients initially presented with gastrointestinal symptoms (4). SARS-CoV-2 RNA could be detected in fecal samples from 53.42% of patients (5). This indicates that SARS-CoV-2 probably can be transmitted by the fecal-oral route (6, 7). However, few studies report the transmission of SARS-CoV-2 during breastfeeding. Whether SARS-CoV-2 can be transmitted from mother to their breast-fed babies via breastmilk is still unknown. In this study, we report a breastfeeding woman who was infected with SARS-CoV-2 and diagnosed with COVID-19 presenting with gastrointestinal symptoms with persistent SARS-CoV-2 RNA positivity in her feces but negativity in her breastmilk. She bottle fed her baby with her breastmilk after treatment. The baby seems healthy and unaffected after a 1-month follow up.

## Case Report

On January 24, 2020, a 30-year-old woman started to have changes of bowel habits. She used to have a bowel movement once a day with Bristol 3 feces. Now, she had a bowel movement 1–2 times per day with Bristol 4 and 5 feces, with increased borborygmi and urgency of defecation. Two days later (on January 26), she began to have fever at noon with a maximum body temperature of 37.8°C. Some tests were performed for her on January 30, 2020 to work up the cause of her fever. The blood routine examination showed that the count of lymphocytes was 0.54 × 10^9^/L with a relative ratio of 11.1%, which was below the normal range (Supplementary Table 1). The level of high sensitive C reaction protein (hsCRP) was 5.87 mg/L, which was a little higher than the normal range (Supplementary Table 1). Even without any respiratory symptoms, she underwent a chest computerized tomography (CT) scan because of a history of recent contact with a COVID-19 patient. The CT scan reported as showing no abnormalities. Oropharyngeal swabs were tested positive for 2019-nCoV using real-time reverse-transcriptase–polymerase-chain-reaction (RT-PCR) which was performed by using a SARS-CoV-2 nucleic acid detection kit (Shanghai bio-germ Medical Technology Co) to detect the 2019-nCoV ORF1ab and N gene with a cycle threshold (Ct) value of 38 or less was defined as a positive on February 7, 2020 (Table 1). The extraction and detection methods were performed according to the manufacturer's protocol. Her body temperature fluctuated from 36.3 to 37.5°C after she took umifenovir (arbidol hydrochloride) 200 mg orally twice a day, which is an antiviral to treat influenza in China. She was admitted to the Union Hospital on February 9, 2020 with the diagnosis of COVID-2019. The patient has not reported any respiratory symptoms since January 24.

**Table 1:** Results of RT-PCR Testing for the SARS-CoV-2.

	**Oropharyngeal swabs**	**Fecal samples**	**Breastmilk samples**
Illness day 15	Positive	NT	NT
Illness day 16	NT	NT	NT
Illness day 17	NT	NT	NT
Illness day 18	NT	NT	NT
Illness day 19	NT	NT	NT
Illness day 20	Negative	NT	NT
Illness day 21	Negative	NT	NT
Illness day 22	NT	Positive	NT
Illness day 23	Negative	NT	NT
Illness day 24	NT	NT	Negative
Illness day 25	NT	NT	Negative
Illness day 26	NT	Positive	NT
Illness day 27	NT	NT	NT
Illness day 28	NT	NT	NT
Illness day 29	NT	Positive	NT

*“NT” represents samples were not tested. RT-PCR, Real-Time Reverse Transcriptase Polymerase Chain Reaction; SARS-CoV-2, severe acute respiratory syndrome coronavirus 2*.

The patient stated that she was living in Wuhan, and two of her family members living with her were confirmed to be infected with SARS-CoV-2 on January 27 and January 30, 2020, respectively. Apart from a history of cesarean section to deliver an infant on January 16, 2020, the patient was an otherwise healthy non-smoker. The physical examination revealed a body temperature of 36.8°C, blood pressure of 120/87 mmHg, pulse of 90 beats per minute, respiratory rate of 14 breaths per minute, and subcutaneous oxygen saturation (SpO2) of 98%.

Blood routine examination showed that the count of lymphocyte increased to 0.9–1.3 × 10^9^/L during hospitalization (Supplementary Table 1). A rapid nucleic acid amplification test (NAAT) for influenza A and B was negative. The antibodies of mycoplasma, chlamydia, Respiratory Syncytial Virus (RSA), adenovirus, and coxsackie virus were tested negative in her serum. In serum, the levels of D-dimer, alanine aminotransfease (ALT), aspartate transaminase (AST), lactic dehydrogenase (LDH), creatinine (Cr), and blood urea nitrogen (BUN) were in normal range (Supplementary Table 1). She repeated chest CT scans on February 7 and February 22, respectively, which were basically normal, showing no signs of viral pneumonia. She was treated with aerosolized interferon α2β from February 9, 2020.

**Figure 1:**
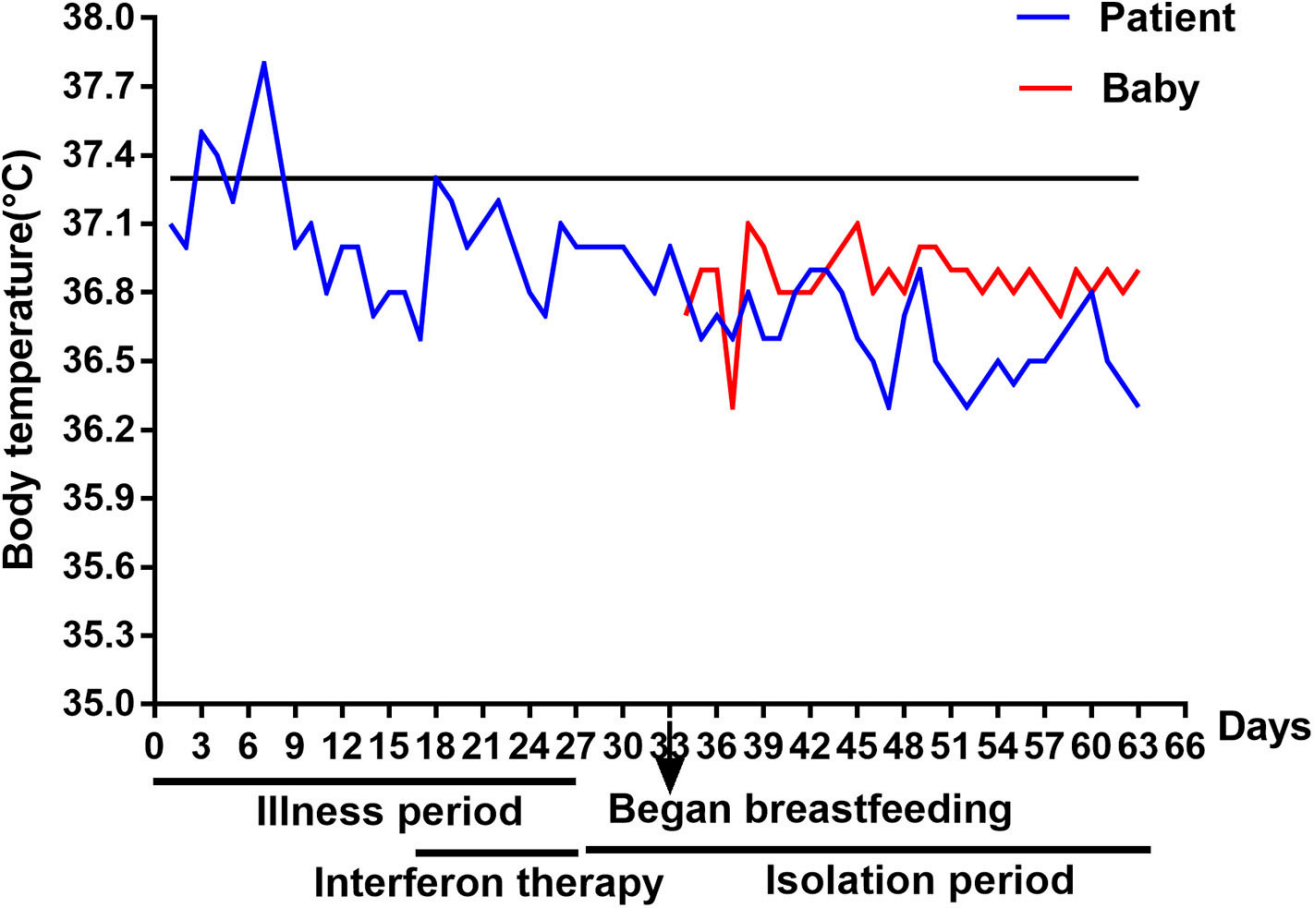
Change of body temperature for the patient and her baby. The solid black line represents the lower limit of fever for subjects. The body temperature for the patient is higher from the 3rd day to the 8th day. The body temperature for the patient was in normal range during the following days including the breastfeeding period. And, the body temperature for the breast-fed baby was also in normal range during the 1-month follow up.

Oropharyngeal swabs tests for SARS-CoV-2 were performed at the fourth and 5th days of the interferon treatment, and both of the results were negative. Because of her gastrointestinal symptoms, she was requested to have her stool examined. The stool routine test was normal, and no parasite eggs or fungi were detected, while fecal samples were persistently positive for SARS-CoV-2 RNA (Table 1). After taking the probiotics, *Saccharomyces boulardii* Sachets, her urgency of defecation subsided. Thus, she was discharged from the hospital after treatment and 1 week later, she began to bottle feed her baby with her breastmilk as breastmilk samples were tested negative for SARS-CoV-2 RNA (Table 1), and the IgG antibody of SARS-CoV-2 was tested positive in her serum. Since her fecal samples were positive for SARS-CoV-2 RNA, the patient did not contact her baby directly. She pumped the breastmilk, and her unaffected family member helped to feed the baby. The baby boy seems healthy during the following 1-month follow up, whose body temperature (Figure 1), consciousness, and growth were normal without any symptoms. The baby is still actively monitored by his family.

## Discussion

On the basis of previous reports from China, the most common transmission route of SARS-CoV-2 among subjects are respiratory droplets and contact, and fecal-oral route maybe one of the transmission routes (3). We have described a COVID-19 patient who was bottle feeding her baby with breastmilk without transmission. SARS-CoV-2 was not detected in her breastmilk at the time when her stool samples tested positive for SARS-CoV-2. The baby seems unaffected during the 1-month follow up. This indicated that it may be safe for a COVID-19 patient to feed her baby using breastmilk.

Most COVID-19 patients presented with cough and low-grade intermittent fevers with ground-glass opacity on chest CT (8). While some patients initially presented with gastrointestinal symptoms, such as diarrhea and vomiting (4, 8). We have reported a COVID-19 patient initially presenting with change of bowel habits and increased borborygmi. These non-specific symptoms of mild illness in the clinical course of SARS-CoV-2 infection may be indistinguishable clinically from many other common gastrointestinal diseases. It is easy to be ignored by the patients. Key aspects of this case included the decision made by the patient to seek medical attention after recognition of the history of contacts with COVID-19 patients and prompt isolation and treatment of the patient. The mechanism that COVID-19 patients present with gastrointestinal symptoms is unclear. It was reported that SARS-CoV-2 uses angiotensin converting enzyme II (ACE2) for cell entry (9). Apart from the lung alveolar epithelial cells, enterocytes of the small intestine also express ACE2 (10). And, indeed, SARS-CoV-2 can enter enterocytes in the gastrointestinal tract and can possibly cause symptoms (5).

Detection of SARS-CoV-2 RNA in specimens from the upper respiratory tract suggests the potential transmissibility through droplets from the respiratory tract. Interestingly, we also detected SARS-CoV-2 RNA in stool samples from this patient. Several studies also reported that SARS-CoV-2 RNA could be detected from fecal samples of COVID-19 patients (4, 5). SARS-CoV-2 was also isolated from the mucus of intestine and esophagus (8). It was reported that the middle east respiratory syndrome (MERS) coronavirus could be detected in fecal samples from MERS patients (11). Further study confirmed that the middle east respiratory syndrome coronavirus could be transmitted through the fecal-oral route in the animal model (12). These indicated that the fecal-oral route may be the potential transmission route of SARS-CoV-2.

SARS-CoV-2 RNA was not detected in the breastmilk of our patient, which is consistent with another report (13). While other studies reported that SARS-CoV-2 RNA was detected in breastmilk for some cases with live viruses being undetected or not assessed (14–16). This maybe related with a low expression of ACE2 in breast (17). Moreover, there is no evidence showing that coronavirus can be transmitted via breastmilk in SARS or MERS cases (18, 19). In our study, the baby seems unaffected during breastfeeding. This indicates that SARS-CoV-2 probably is rarely transmitted through human milk. In this case, SARS-CoV-2 IgG antibody in serum of the patients may result in passive immunity during breastfeeding. Human milk testing of antibody from the patients and serum testing from the baby may provide a better understanding of the immune response to SARS-CoV-2 infection during feeding of human milk.

Limitations of this study was that we only observed clinical symptoms of the baby and did not test SARS-CoV-2 RNA or antibody from the baby. The baby may be asymptomatic despite being infected (20), which is probably related with the blunted immune response toward the SARS-CoV infection in children (21). Data on larger numbers of breastfeeding women infected with SARS-CoV-2 may help define infection risks and find prevention strategies.

In summary, this report, in conjunction with the reports from China, provides an initial view of the spectrum of illness and outcomes associated with breastfeeding-related SARS-CoV-2 infection. Multiple factors might contribute to this outcome, such as differences in host immune response, in the incubation period, and the presence of coexisting conditions.

Data on larger numbers of breastfeeding women infected with SARS-CoV-2 and long-term follow-up of babies are needed to fully understand the SARS-CoV-2 infection during breastfeeding. This will eventually provide a solid basis for clinical guidelines to manage future cases.

## Data Availability Statement

The original contributions presented in the study are included in the article/Supplementary Materials, further inquiries can be directed to the corresponding author/s.

## Ethics Statement

The studies involving human participants were reviewed and approved by The institutional board of Union Hospital, Huazhong University of Science and Technology (20200033). Affiliation: Union Hospital, Huazhong University of Science and Technology. The patients/participants provided their written informed consent to participate in this study. Written informed consent was obtained from the individual(s) for the publication of any potentially identifiable images or data included in this article.

## Author Contributions

HC was responsible for the acquisition, analysis, interpretation of data, and drafting of the manuscript. JL and JY provided assistance in data acquisition critical revision of the manuscript. TB provided assistance in data analysis. BS and LZ provided critical revision of the manuscript. LY and XH were responsible for the study concept and design, critical revision of the manuscript, and study supervision. All authors contributed to the article and approved the submitted version.

## Conflict of Interest

The authors declare that the research was conducted in the absence of any commercial or financial relationships that could be construed as a potential conflict of interest.
