# Supplementary material for: Persistent SARS-CoV-2 RNA Positive in Feces but Negative in Breastmilk: A Case Report of COVID-19 in a Breastfeeding Patient
Source: Front Med (Lausanne). 2020 Dec 2;7:562700. doi: 10.3389/fmed.2020.562700 (PMC7738631; doi:10.3389/fmed.2020.562700)
Supplement: Supplementary file 1 [file Data_Sheet_1.pdf]

Persistent SARS-CoV-2 RNA Positive in faeces but negative in breastmilk: A case report of COVID-19 in a breastfeeding patient

Huikuan Chu[#1], Jing Li[#1], Jingjing Yan[#1], Tai Bai[1], Bernd Schnabl[2], Li Zou[3], Ling Yang[*1], Xiaohua Hou[*1]

[1]Division of Gastroenterology, Union Hospital, Tongji Medical College, Huazhong University of Science and Technology, 1277 Jiefang Avenue, Wuhan 430022, China;

[2]Department of Medicine, University of California San Diego, La Jolla, CA, USA;

[3]Department of *Obstetrics* & *Gynecology,* Union Hospital, Tongji Medical College, Huazhong University of Science and Technology, 1277 Jiefang Avenue, Wuhan 430022, China

[#] These authors contributed equally to this paper.

*** Correspondence to:**

Ling Yang, M.D., Ph.D. Division of Gastroenterology, Union Hospital, Tongji Medical College, Huazhong University of Science and Technology, 1277 Jiefang Avenue, Wuhan, 430022, China.

Email: hepayang@163.com;

phone +86-2785726678, +8613971178791

Xiaohua Hou, M.D., Ph.D. Division of Gastroenterology, Union Hospital, Tongji Medical College, Huazhong University of Science and Technology, 1277 Jiefang Avenue, Wuhan, 430022, China.

Email: houxh@hust.edu.cn

Phone +86 -2785726678, +8613035143646

Supplementary Table 1. Laboratory results for patients.

				Admission day 2	Admission day 4	Admission day 5	Admission day 8	Admission day 11	Admission day 14
	normal range	Illness day 7	Illness day 15	Illness day 18	Illness day 20	Illness day 21	Illness day 24	Illness day 27	Illness day 30
White blood cell count (×10^9 /L)	3.5-9.5	4.84	5.19	4.51	4.76	5.74	4.82	5.35	4.78
Neutrophil count (×10^9 /L)	1.8-6.3	4.00	3.47	3.27	2.99	4.10	3.18	3.51	3.10
Lymphocyte count (×10^9 /L)	1.1-3.2	0.54	1.29	0.90	1.22	1.10	1.09	1.23	1.10
Lymphocyte (%)	20-50	11.10	24.80	20.00	25.60	19.20	22.60	23.00	22.50
Hemoglobin (g/L)	130-175	140	143	136	122	129	124	120	123
Platelet count (×10^9 /L)	125-350	217	228	215	218	220	187	207	189
hsCRP (mg/L)	<4.0	5.87	2.34	<3.14	—	<3.14	<3.14	4.41	4.17
Albumin (g/L)	35-55	—	47	47.3	—	42	42.6	40.3	41.8
Aspartate aminotransferase (U/L)	8-40	—	18	19	—	21	19	20	25
Alanine aminotransferase (U/L)	5-40	—	16	17	—	13	13	13	19
Lactic dehydrogenase (U/L)	109-245	—	200	220	—	191	186	168	197
Creatinine (umol/L)	0.5-1.5	—	0.81	0.74	—	0.71	0.75	0.63	0.64
Blood urea nitrogen (mmol/L)	2.9-8.2	—	3.5	3.71	—	4.28	4.86	5.13	5.05
D-dimer (mg/L)	<0.5	—	—	1.12	—	—	—	—	—
